# Differences between fellow eyes of acute and chronic primary angle closure (glaucoma): An ultrasound biomicroscopy quantitative study

**DOI:** 10.1371/journal.pone.0193006

**Published:** 2018-02-15

**Authors:** Mengwei Li, Yuhong Chen, Xiaoxiao Chen, Wenqing Zhu, Xueli Chen, Xiaolei Wang, Yuan Fang, Xiangmei Kong, Yi Dai, Junyi Chen, Xinghuai Sun

**Affiliations:** 1 Department of Ophthalmology and Visual Science, Eye, Ear, Nose and Throat Hospital, Shanghai Medical College of Fudan University, Shanghai, China; 2 Key Laboratory of Myopia, Ministry of Health (Fudan University) and Key Laboratory of Visual Impairment and Restoration of Shanghai, Shanghai, China; 3 State Key Laboratory of Medical Neurobiology, Institutes of Brain Science and Collaborative Innovation Center for Brain Science, Fudan University, Shanghai, China; University of Sydney, AUSTRALIA

## Abstract

**Purpose:**

To compare various biometric parameters between fellow eyes of acute primary angle closure (glaucoma) [APAC(G)] and fellow eyes of chronic primary angle closure (glaucoma) [CPAC(G)].

**Methods:**

Ultrasound biomicroscopy examinations were performed on 47 patients with unilateral APAC(G) and 41 patients with asymmetric CPAC(G) before laser peripheral iridotomy and pilocarpine treatment. Anterior chamber depth and width (ACD and ACW), lens vault (LV), iris curvature (IC), iris root distance (IRD), trabecular-ciliary process distance (TCPD), iris-ciliary process distance (ICPD), trabecular-ciliary angle (TCA), and other biometric parameters were compared between fellow eyes of APAC(G) and fellow eyes of CAPC(G).

**Results:**

Compared with fellow eyes of CPAC(G), fellow eyes of APAC(G) had smaller ACD (*P* < 0.001), ACW (*P* = 0.007), TCPD (*P* = 0.016), ICPD (*P* = 0.008), and TCA (*P* = 0.006), as well as larger LV (*P* = 0.002), IC (*P* = 0.012), and IRD (*P* = 0.003). On multivariate logistic regression analyses, a 0.1 mm decrease in ACD (odds ratio [OR]: 0.705, 95%CI: 0.564–0.880, *P* = 0.002), ICPD (OR: 0.557, 95%CI: 0.335–0.925, *P* = 0.024), and a 0.1 mm increase in IRD (OR: 2.707, 95%CI: 1.025–7.149, *P* = 0.045), was significantly associated with occurrence of acute angle closures.

**Conclusions:**

Fellow eyes of APAC(G) had smaller anterior segment dimensions, higher LV, more posterior iris insertion, greater IC, and more anteriorly rotated ciliary body compared with fellow eyes of CPAC(G). ACD, ICPD, and IRD were the three most important parameters that distinguish eyes predisposed to APAC(G) or CPAC(G).

## Introduction

Primary angle closure disease has greater prevalence in East Asian countries, especially in China, than that in western countries [1,2]. This potentially devastating disease is characterized by appositional approximation or contact between peripheral iris and trabecular meshwork, which can cause two main clinical manifestations: an acute attack or a chronic form [2,3]. Characteristic anatomic factors are associated with both forms of angle closure, such as short axial length (AXL), shallow anterior chamber depth (ACD), small anterior chamber width (ACW), thick iris with greater curvature, and increased lens vault (LV) [4–6]. However, differences of anatomic structures remain to be clarified between acute form and chronic form.

With the advent of ophthalmic imaging techniques such as anterior segment optical coherence tomography (AS-OCT) and ultrasound biomicroscopy (UBM), numerous reliable insights have been gained into the ocular biometric differences between acute primary angle closure (glaucoma) [APAC(G)] and chronic primary angle closure (glaucoma) [CPAC(G)]. Using AS-OCT, investigators have found that APAC(G) eyes have shallower ACD [7–9], greater LV [7,8,10], and thicker peripheral iris [7] than CPAC(G) eyes. Compared with AS-OCT, the greatest advantage of UBM is its ability to reveal details of structures posterior to the iris. With the help of UBM, researchers have revealed that APAC(G) eyes have not only shallower ACD and more anterior lens position [11], but also shorter trabecular-ciliary process distance (TCPD) [11,12].

However, the appearance of iris atrophy in APAC(G) eyes or extensive peripheral anterior synechia (PAS) in CPAC(G) eyes would affect the measurement of biometric parameters, which might not represent the initial characteristics of anatomic structures before the disease develops [2]. On the other hand, primary angle closure disease has been essentially described as a bilateral condition [2,13]. The risk of undergoing an acute attack in the fellow eye of APAC, if left untreated, has been reported to be about 40% to 80% over five to ten years [13,14]. Also, a proportion of patients diagnosed advanced CPACG in one eye have no PAS or only mild PAS in the fellow eye, which would gradually develop glaucoma as well, mostly in the same form as the advanced eye [2]. Therefore, the fellow eyes of unilateral APAC(G) and asymmetric CPAC(G) could, to some degree, reflect the anatomic configuration of the severely affected eyes due to the high similarities between two eyes in the same person [15]. Factors that make these predisposed eyes develop APAC(G) or CPAC(G) are currently unknown. To our knowledge, only one study compared biometric features in fellow eyes of APAC and CPACG by using UBM, which concluded that the fellow eyes of CPACG had deeper ACD, thicker basal iris, and more anteriorly rotated ciliary process than the fellow eyes of APAC [16]. In that study, however, patients were evaluated only after laser peripheral iridotomy (LPI), thus pupillary block component could not be assessed due to significant alterations in the anterior segment morphology. Besides, many important parameters such ACW and LV were not measured in that study.

This prospective UBM quantitative study comprehensively compare various parameters between fellow eyes of unilateral APAC(G) and fellow eyes of asymmetric CPAC(G) before LPI and pilocarpine treatment to identify the differences of anatomic structures in these two forms of angle closure diseases.

## Methods

This prospective, cross-sectional study was conducted at the Eye and Ear Nose and Throat Hospital of Fudan University (Shanghai, China). The study followed the tenets of the declaration of Helsinki and was approved by the human subjects review committee of the Eye and Ear Nose and Throat Hospital of Fudan University in Shanghai, China. Written informed consents were obtained for all the patients.

The patients diagnosed with unilateral APAC(G) and asymmetric CPAC(G) were recruited from the Glaucoma Clinic in our hospital from Mar. 2015 to Dec. 2016. All the patients were referred from the Outpatient Department and Emergency Department and then underwent examinations of low-coherence interferometry and UBM examination immediately before therapeutic interventions.

### Participants

All unilateral APAC(G) and asymmetric CPAC(G) patients included had not undergone LPI or intraocular surgery. Pilocarpine was never used or discontinued for at least one week in the fellow eyes of APAC(G) and CPAC(G) at the time of consultation.

The affected eyes of unilateral APAC(G) were defined by the following criteria: (1) presence of two of the following symptoms: headache, nausea and/or vomiting, ocular or periocular pain, and blurred vision with haloes; (2) presenting Intraocular pressure (IOP) >30mmHg; (3) presence of at least three of the following signs: conjunctival injection, corneal epithelial edema, shallow anterior chamber, and mid-dilated pupil. The fellow eyes of APAC(G) had never experienced an acute attack and had narrow angle, but no PAS or less than three cumulative clock-hours PAS.

The relatively more severe eyes of asymmetric CPAC(G) were defined as: (1) absence of symptoms of an acute attack; (2) absence of signs of a prior acute attack; (3) gonioscopically confirmed PAS of more than at least three cumulative clock-hours; (4) presenting IOP >21mmHg; (5) along with normal optic disk and visual field (CPAC) or glaucomatous optic neuropathy or visual field defect (CPACG). The fellow eyes of CPAC(G) had no PAS or less than three cumulative clock-hours PAS.

The fellow eyes of APAC(G) and CPAC(G) with the presence of appositional contact between the peripheral iris and the posterior trabecular meshwork but without PAS, raised IOP or glaucomatous optic neuropathy were defined as primary angle closure suspect (PACS). The eyes with the presence of iridotrabecular contact and an elevated IOP or PAS with no secondary cause for the PAS, but without glaucomatous optic neuropathy were defined as primary angle closure (PAC). Primary angle closure glaucoma (PACG) was defined as eyes with PAC accompanied with glaucomatous optic neuropathy [2].

The exclusion criteria were: (1) secondary angle closure, such as iris neovascularization, lens intumescence, or subluxation and uveitis; (2) plateau iris configuration, which is defined as an immediate anterior bend of the iris in the far periphery of the iris and a flat, unbowed iris extending towards the pupil from that sharp peripheral bend by gonioscopy [17]; (Plateau iris configuration was excluded because of its unique biometric structures different from other primary angle closures.) (3) previous laser or intraocular surgery; (4) patients with axial length < 19mm; (5) other eye disorders which could potentially affect biometric parameters such as macular degeneration and retinal detachment.

### Ophthalmic examination

All patients underwent a comprehensive ophthalmic examination including detailed silt-lamp examination of anterior segment, and stereoscopic evaluation of the optic disk using a 90-diopter lens (Volk Optical, Inc., Mentor, OH, USA). Gonioscopy was performed with a 4-mirror goniolens (Volk Optical, Inc., Mentor, OH, USA) with and without indentation under dark conditions. The angle was defined as closed if PAS exist when the posterior trabecular meshwork was not visible under cornea indentation [18]. Intraocular pressure was measured with Goldmann applanation tonometry. Low-coherence interferometry (LenStar 900; Haag-Streit, Koeniz, Switzerland) was used to determine AXL, central corneal thickness (CCT), lens thickness (LT), flat keratometry (Kf) and steep keratometry (Ks).

### Ultrasound biomicroscopy and analysis

The UBM (MD-300L; MEDA Co., Ltd., Tianjin, China) measurements were performed before therapeutic interventions by one of two experienced operators (XC and JC) who were masked to the clinical data. The frequency of the probe transducer was 50 MHz. Patients were examined in a supine position in room light (illumination about 120 lux, measured with an luminance meter [model DT-1301, Everbest Machinery Industry Co., Ltd, Shenzhen, China]). After topical anesthesia was applied, an eye cup containing hydroxyethyl cellulose and physiologic saline was mounted on the globe, and the transducer was applied gently to the limbal area with care to avoid compression on the globe. The measurements were obtained at the 12 (superior), 3 (nasal), 6 (inferior), and 9 (temporal) o’clock positions of both eyes of each patient. If PAS was right at 3, 6, 9, and 12 o’clock according to the gonioscopy, the position would be avoided for measurement and the picture next to the PAS position will be used instead. Patients would be excluded if scleral spurs were blurry. Scans were also centered on the pupil and taken along 3–9 (nasal-temporal) o’clock to obtain full view of the anterior segment.

**Fig 1:**
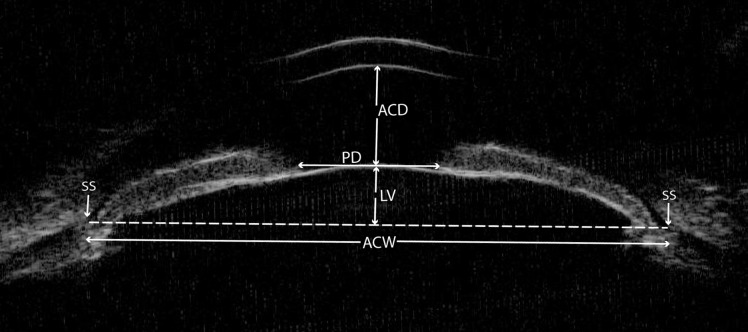
The determination of the parameters on an ultrasound biomicroscopy image of the horizontal perpendicular full view scans at the nasal-temporal position centered over the pupil. ACD = anterior chamber depth; ACW = anterior chamber width; LV = lens vault; PD = pupil diameter; SS = scleral spur.

**Fig 2:**
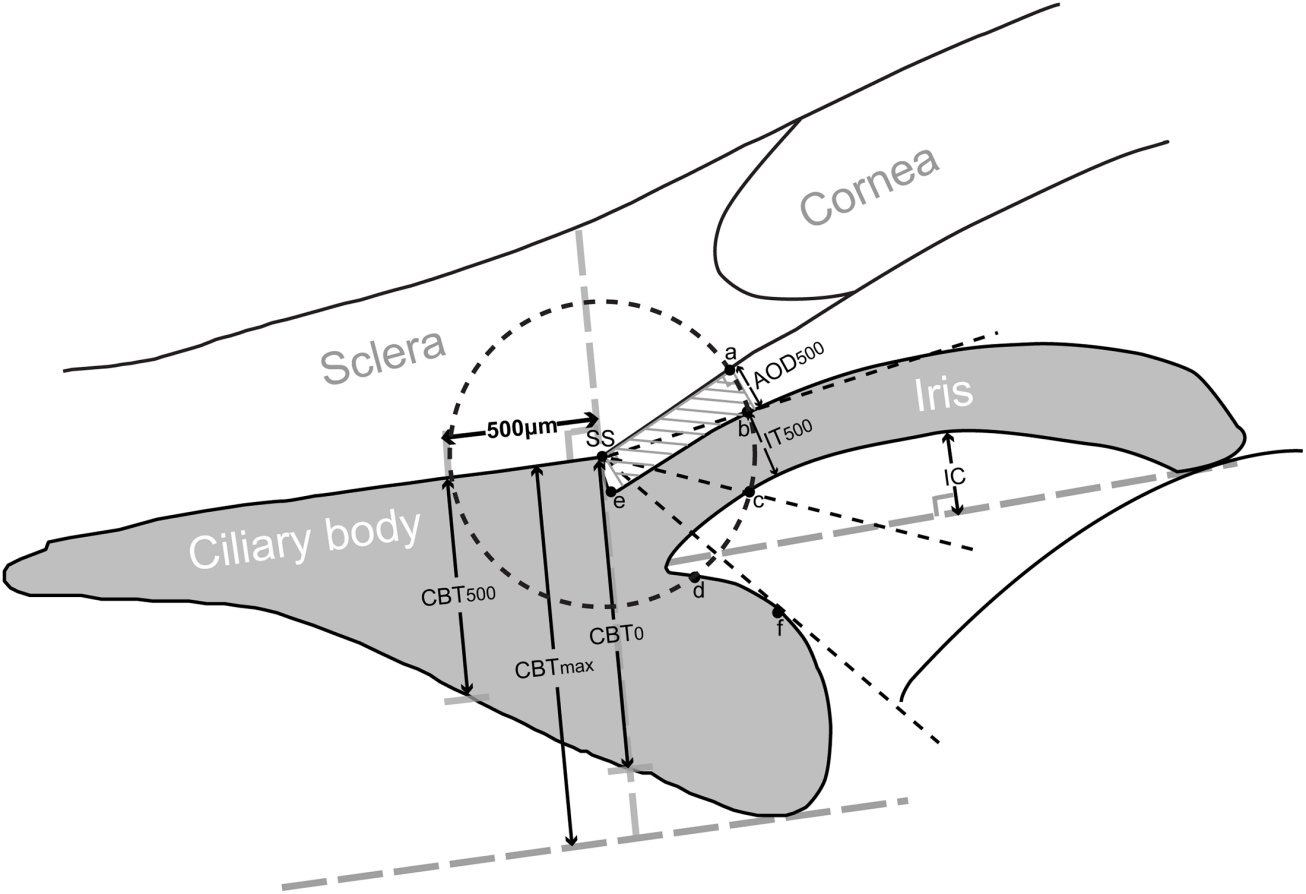
The determination of the parameters on an ultrasound biomicroscopy diagram of the radial scans centered over the limbus. A circle with a radius of 500 μm centered on the scleral spur (SS) is drawn. Angle-opening distance at 500 μm (AOD500) is the distance between the posterior corneal surface and the anterior iris surface on a line perpendicular to the trabecular meshwork 500 μm from the SS. Trabecular-iris space area at 500 μm (TISA500) is the area bounded anteriorly by AOD500 as determined, posteriorly by a line drawn from the SS perpendicular to the plane of the inner scleral wall to the iris, superiorly by the inner corneoscleral wall, and inferiorly by the iris surface. Trabecular-anterior iris surface angle (TAIA) is the angle between the posterior corneal surface and the anterior iris surface (angle of “a-SS-b”). Trabecular-posterior iris surface angle (TPIA) is the angle between the posterior corneal surface and the posterior iris surface (angle of “a-SS-c”). Iris thickness at 500 μm (IT500) is iris thickness at 500 μm from the SS. Iris curvature (IC) is the perpendicular distance from a line between the most central to the most peripheral points of the iris pigment epithelium to the posterior iris surface at the point of greatest convexity. Iris root distance (IRD): the distance from the SS to the insertion location of the iris into the ciliary body (line of “SS-e”). Trabecular-ciliary process distance (TCPD) is a line extending from the corneal endothelium 500 μm anterior to the SS toward the ciliary processes (line of “ad”). Iris-ciliary process distance (ICPD) is the posterior surface of the iris 500 μm anterior to the SS toward the ciliary processes (line of “cd”). Trabecular-ciliary angle (TCA) is the angle between the posterior corneal surface and the anterior surface of the ciliary body (angle of “a-SS-f”). Maximum ciliary body thickness (CBTmax) is the distance from the most inner point of the ciliary body to the inner wall of sclera or its extended line. Ciliary body thicknesses at the point of the SS (CBT0) and at a distance of 500 μm (CBT500) are also measured.

All captured UBM images of fellow eyes of APAC(G) and CPAC(G) were analyzed using the built-in software in the machine by a single observer (ML), masked to clinical data. The anterior segment parameters evaluated in this UBM quantitative study were based on the traditional parameters developed by Pavlin et al. [19,20] and the new defined parameters described in recent studies [5,6,21–23]. From the horizontal perpendicular full view scans at the nasal-temporal position centered over the pupil, the following parameters were measured (Fig 1): (1) ACD: the axial distance between the corneal endothelium and the anterior lens surface [24]; (2) pupil diameter (PD): the shortest distance between the pupil edges of the iris cross-sections; (3) ACW: the distance between the two scleral spurs [6]; (4) LV: the perpendicular distance from the anterior pole of the lens to the horizontal line between the scleral spurs [5]. From the radial scans at the superior, nasal, inferior, and temporal positions centered over the limbus, the following parameters were measured (Fig 2): (1) angle-opening distance at 500 μm (AOD500): the distance between the posterior corneal surface and the anterior iris surface on a line perpendicular to the trabecular meshwork 500 μm from the scleral spur [8]; (2) trabecular-iris space area at 500 μm (TISA500): the area bounded anteriorly by AOD500 as determined, posteriorly by a line drawn from the scleral spur perpendicular to the plane of the inner scleral wall to the iris, superiorly by the inner corneoscleral wall, and inferiorly by the iris surface [8]; (3) trabecular-anterior iris surface angle (TAIA): the angle between the posterior corneal surface and the anterior iris surface [23]; (4) trabecular-posterior iris surface angle (TPIA): the angle between the posterior corneal surface and the posterior iris surface [23]; (5) iris thickness at 500 μm (IT500): iris thickness at 500 μm from the scleral spur [25]; (6) iris curvature (IC): the perpendicular distance from a line between the most central to the most peripheral points of the iris pigment epithelium to the posterior iris surface at the point of greatest convexity [25]; (7) iris root distance (IRD): the distance from the scleral spur to the insertion location of the iris into the ciliary body [26]; (8) TCPD: a line extending from the corneal endothelium 500 μm anterior to the scleral spur toward the ciliary processes [22]; (9) iris-ciliary process distance (ICPD): the posterior surface of the iris 500 μm anterior to the scleral spur toward the ciliary processes [22]; (10) trabecular-ciliary angle (TCA): the angle between the posterior corneal surface and the anterior surface of the ciliary body [21]; (11) maximum ciliary body thickness (CBTmax): the distance from the most inner point of the ciliary body to the inner wall of sclera or its extended line [21]; (12) ciliary body thickness at the point of the scleral spur (CBT0) and at a distance of 500 μm (CBT500) [21]. For each parameter, the mean of three measurements with the same image was used as the final value.

### Repeatability and reproducibility

Since the poor reproducibility is one of shortcomings of UBM for measuring angle structures compared with AS-OCT [27], we did repeatability and reproducibility analyses of our UBM parameters. The repeatability and reproducibility of UBM measurements were assessed in a random subset of 15 images from angle-closure patients included. A single observer (ML) measured each image twice within two-week interval to decide intra-observer variability. Besides, a second observer (XC) measured the same images independently on a different day to decide inter-observer variability. The coefficient of the intra-class correlation (ICC) was used to calculate the intra-observer and inter-observer variability.

### Statistical analysis

Statistical analyses were performed using SPSS version 20.0 (SPSS, Inc., Chicago, IL, USA). The averages and standard deviations were calculated for continuous data and the frequency distribution was used for categorical data. The averages of the superior, nasal, inferior, and temporal AOD500, TISA500, TAIA, TPIA, IT500, IC, IRD, TCPD, ICPD, TCA, CBT0, CBTmax, and CBT500 were calculated and used for final analyses. Independent *t* tests were used to compare the continuous variables between fellow eyes of APAC(G) and fellow eyes of CPAC(G). Chi-square tests were used to compare the categorical variables. Univariate and multivariate logistic regression analyses after adjusting for age and sex were performed to identify the most important parameters differentiating APAC(G) and CPAC(G). Variables with *P* < 0.05 and variance inflation factor < 5 in the univariate outcome were incorporated in the forward multivariate logistic regression model. A *P* value less than 0.05 was considered statistically significant.

## Results

A total of 91 consecutive patients who presented with unilateral APAC(G) and asymmetric CPAC(G) were recruited, of which three patients were excluded due to difficulty in determining scleral spurs. Accordingly, 47 patients with unilateral APAC(G) and 41 patients with asymmetric CPAC(G) were included in the final analysis. Of the 47 fellow eyes of APAC(G), 39 were diagnosed as PACS and 8 as PAC; of the 41 fellow eyes of CPAC(G), 25 were diagnosed as PACS and 16 as PAC. There were no significant differences in age, sex, IOP, medication numbers, AXL, CCT, LT, or keratometry between fellow eyes of APAC(G) and fellow eyes of CPAC(G). The C/D ratio in fellow eyes of CPAC(G) was significantly larger than that of APAC(G) (*P* = 0.035), and the fellow eyes of CPAC(G) had a wider scope of angle closure on gonioscopy (*P* = 0.014) (Table 1).

**Table 1:** Demographics, clinical features and basic biometric parameters measured by low-coherence interferometry in fellow eyes of acute primary angle closure (Glaucoma) and chronic primary angle closure (Glaucoma).

Parameters	Fellow Eyes of APAC(G)	Fellow Eyes of CPAC(G)	*P* Value
No. eyes	47	41	-
Age (yrs)	65.3 ± 9.4	62.8 ± 10.6	0.240
Sex (M/F)	11/36	17/24	0.070*
IOP (mmHg)	16.06 ± 3.27	17.41± 3.63	0.070
C/D ratio	0.37 ± 0.10	0.42 ± 0.11	**0.035**
Medication number	0.06 ± 0.32	0.15 ± 0.48	0.340
Closed angles (clock-hours)	0.96 ± 1.41	1.68 ± 1.27	**0.014**
Diagnosis (PACS/PAC)	39/8	25/16	0.021*
Axial length (mm)	22.30 ± 0.99	22.60 ± 0.68	0.106
CCT (μm)	537.23 ± 34.33	547.07 ± 29.90	0.158
Lens thickness (mm)	4.83 ± 0.45	4.67 ± 0.40	0.085
Flat keratometry (D)	43.99 ± 1.66	44.03 ± 1.50	0.909
Steep keratometry (D)	44.96 ± 1.66	44.64 ± 1.61	0.371

* Chi-square test

Bold values are *P* < 0.05. APAC(G) = acute primary angle closure (glaucoma); C/D ratio = cup-disk ratio; CCT = central corneal thickness; CPAC(G) = chronic primary angle closure (glaucoma); PAC = primary angle closure; PACS = primary angle closure suspect; IOP = intraocular pressure.

The intra-observer ICC ranged from 0.948 to 0.999, while the inter-observer ICC ranged from 0.827 to 0.998 (Table 2), which showed good repeatability and reproducibility of all UBM parameters measured in this study.

**Table 2:** Intra-observer and Inter-observer intra-class coefficients of the ultrasound biomicroscopy parameters.

Parameters	Intra-class Coefficients	
	Intra-observer	Inter-observer
ACD	0.999	0.998
PD	0.999	0.995
ACW	0.997	0.994
LV	0.999	0.995
AOD500	0.988	0.980
TISA500	0.948	0.880
TAIA	0.981	0.979
TPIA	0.989	0.977
IT500	0.982	0.932
IC	0.986	0.881
IRD	0.990	0.930
TCPD	0.987	0.827
ICPD	0.990	0.931
TCA	0.986	0.959
CBT0	0.996	0.915
CBTmax	0.997	0.867
CBT500	0.996	0.915

ACD = anterior chamber depth; ACW = anterior chamber width; AOD500 = angle-opening distance at 500 μm; CBT0 = ciliary body thickness at the point of the scleral spur; CBT500 = ciliary body thickness at a distance of 500 μm; CBTmax = maximum ciliary body thickness; IC = iris curvature; ICPD = iris-ciliary process distance; IRD = iris root distance; IT500 = iris thickness at 500 μm; LV = lens vault; PD = pupil diameter; TAIA = trabecular-anterior iris surface angle; TCA = trabecular-ciliary angle; TCPD = trabecular-ciliary process distance; TISA500 = trabecular-iris space area at 500 μm; TPIA = trabecular-posterior iris surface angle.

The anterior segment UBM parameters of the fellow eyes with APAC(G) and CPAC(G) are compared in Table 3. Fellow eyes of APAC(G) had smaller ACD (*P* < 0.001), ACW (*P* = 0.007), TCPD (*P* = 0.016), ICPD (*P* = 0.008), and TCA (*P* = 0.006), as well as larger LV (*P* = 0.002), IC (*P* = 0.012), and IRD (*P* = 0.003), compared with fellow eyes of CPAC(G). No significant differences were found with regard to PD, AOD500, TISA500, TAIA, TPIA, IT500, CBT0, CBTmax, and CBT500.

**Table 3:** Comparison of ultrasound biomicroscopy parameters in fellow eyes of acute primary angle closure (Glaucoma) and chronic primary angle closure (Glaucoma).

Parameters	Fellow Eyes of APAC(G)	Fellow Eyes of CPAC(G)	*P* Value
ACD (mm)	1.82 ± 0.25	2.01 ± 0.23	<**0.001**
PD (mm)	3.32 ± 0.70	3.39 ± 0.70	0.648
ACW (mm)	11.22 ± 0.46	11.49 ± 0.47	**0.007**
LV (mm)	1.08 ± 0.18	0.96 ± 0.18	**0.002**
AOD500 (mm)	0.062 ± 0.039	0.069 ± 0.047	0.489
TISA500 (mm^2^)	0.030 ± 0.019	0.026 ± 0.017	0.288
TAIA (degree)	7.69 ± 4.10	8.18 ± 5.11	0.620
TPIA (degree)	47.29 ± 7.28	48.17 ± 7.78	0.584
IT500 (mm)	0.355 ± 0.046	0.350 ± 0.050	0.585
IC (mm)	0.318 ± 0.077	0.274 ± 0.086	**0.012**
IRD (mm)	0.088 ± 0.062	0.052 ± 0.048	**0.003**
TCPD (mm)	0.524 ± 0.086	0.570 ± 0.089	**0.016**
ICPD (mm)	0.123 ± 0.096	0.181 ± 0.104	**0.008**
TCA (degree)	58.48 ± 12.33	66.01 ± 12.65	**0.006**
CBT0 (mm)	0.900 ± 0.113	0.913 ± 0.112	0.602
CBTmax (mm)	1.008 ± 0.119	1.014 ± 0.112	0.810
CBT500 (mm)	0.838 ± 0.107	0.859 ± 0.121	0.387

Bold values are *P* < 0.05. ACD = anterior chamber depth; ACW = anterior chamber width; AOD500 = angle-opening distance at 500 μm; APAC(G) = acute primary angle closure (glaucoma); CBT0 = ciliary body thickness at the point of the scleral spur; CBT500 = ciliary body thickness at a distance of 500 μm; CBTmax = maximum ciliary body thickness; CPAC(G) = chronic primary angle closure (glaucoma); IC = iris curvature; ICPD = iris-ciliary process distance; IRD = iris root distance; IT500 = iris thickness at 500 μm; LV = lens vault; PD = pupil diameter; TAIA = trabecular-anterior iris surface angle; TCA = trabecular-ciliary angle; TCPD = trabecular-ciliary process distance; TISA500 = trabecular-iris space area at 500 μm; TPIA = trabecular-posterior iris surface angle.

Univariate logistic regression analyses conformed ACD (*P* = 0.003), ACW (*P* = 0.035), TCPD (*P* = 0.026), ICPD (*P* = 0.011), and TCA (*P* = 0.011) were significantly smaller while LV (*P* = 0.004), IC (*P* = 0.018), and IRD (*P* = 0.013) were significantly larger in fellow eyes of APAC(G) than those in fellow eyes of CPAC(G) with adjusting for age and sex. In the forward multivariate logistic regression, a 0.1 mm decrease in ACD (odds ratio [OR]: 0.705, 95%CI: 0.564–0.880, *P* = 0.002), ICPD (OR: 0.557, 95%CI: 0.335–0.925, *P* = 0.024), and a 0.1 mm increase in IRD (OR: 2.707, 95%CI: 1.025–7.149, *P* = 0.045), was significantly associated with the occurrence of acute attacks (Table 4).

**Table 4:** Relationship of biometric and ultrasound biomicroscopy parameters with presence of acute angle closures.

Parameters	Univariate Logistic Regression			Multivariate Logistic Regression*		
	OR	95% CI	*P* Value	OR	95% CI	*P* Value
AXL (mm)	0.662	0.372, 1.178	0.160			
CCT (μm)	0.993	0.979, 1.007	0.303			
LT (mm)	2.754	0.877, 8.643	0.083			
Kf (D)	0.908	0.684, 1.205	0.503			
Ks (D)	1.073	0.813, 1.416	0.619			
ACD (mm) × 10	0.736	0.602, 0.900	**0.003**	0.705	0.564, 0.880	**0.002**
PD (mm)	0.830	0.444, 1.552	0.560			
ACW (mm)	0.342	0.126, 0.928	**0.035**			
LV (mm)	47.755	3.322, 686.498	**0.004**			
AOD500 (mm) × 10	0.664	0.237, 1.861	0.436			
TISA500 (mm^2^) × 10	2.803	0.218, 36.019	0.429			
TAIA (degree)	0.971	0.882, 1.070	0.551			
TPIA (degree)	0.991	0.934, 1.051	0.991			
IT500 (mm) × 10	1.497	0.594, 3.775	0.393			
IC (mm) × 10	2.171	1.142, 4.128	**0.018**			
IRD (mm) × 10	3.562	1.314, 9.657	**0.013**	2.707	1.025, 7.149	**0.045**
TCPD (mm) × 10	0.549	0.324, 0.931	**0.026**			
ICPD (mm) × 10	0.538	0.333, 0.869	**0.011**	0.557	0.335, 0.925	**0.024**
TCA (degree) ^†^	0.952	0.916, 0.989	**0.011**			
CBT0 (mm)	0.356	0.007, 17.233	0.602			
CBTmax (mm)	0.694	0.016, 30.056	0.849			
CBT500 (mm)	0.153	0.003, 7.216	0.340			

Univariate and multivariate analyses were adjusted for age and sex; bold values are *P* < 0.05.

*Including parameters with *P* < 0.05 in univariate analysis and variance inflation factor less than 5.

^†^ Not included in the multivariate analysis due to multicollinearity with TCPD.

ACD = anterior chamber depth; ACW = anterior chamber width; AOD500 = angle-opening distance at 500 μm; AXL = axial length; CBT0 = ciliary body thickness at the point of the scleral spur; CBT500 = ciliary body thickness at a distance of 500 μm; CBTmax = maximum ciliary body thickness; CCT = central corneal thickness; CI = confidence interval; IC = iris curvature; ICPD = iris-ciliary process distance; IRD = iris root distance; IT500 = iris thickness at 500 μm; Kf = flat keratometry; Ks = steep keratometry; LT = lens thickness; LV = lens vault; OR = odds ratio; PD = pupil diameter; TAIA = trabecular-anterior iris surface angle; TCA = trabecular-ciliary angle; TCPD = trabecular-ciliary process distance; TISA500 = trabecular-iris space area at 500 μm; TPIA = trabecular-posterior iris surface angle.

## Discussion

**Fig 3:**
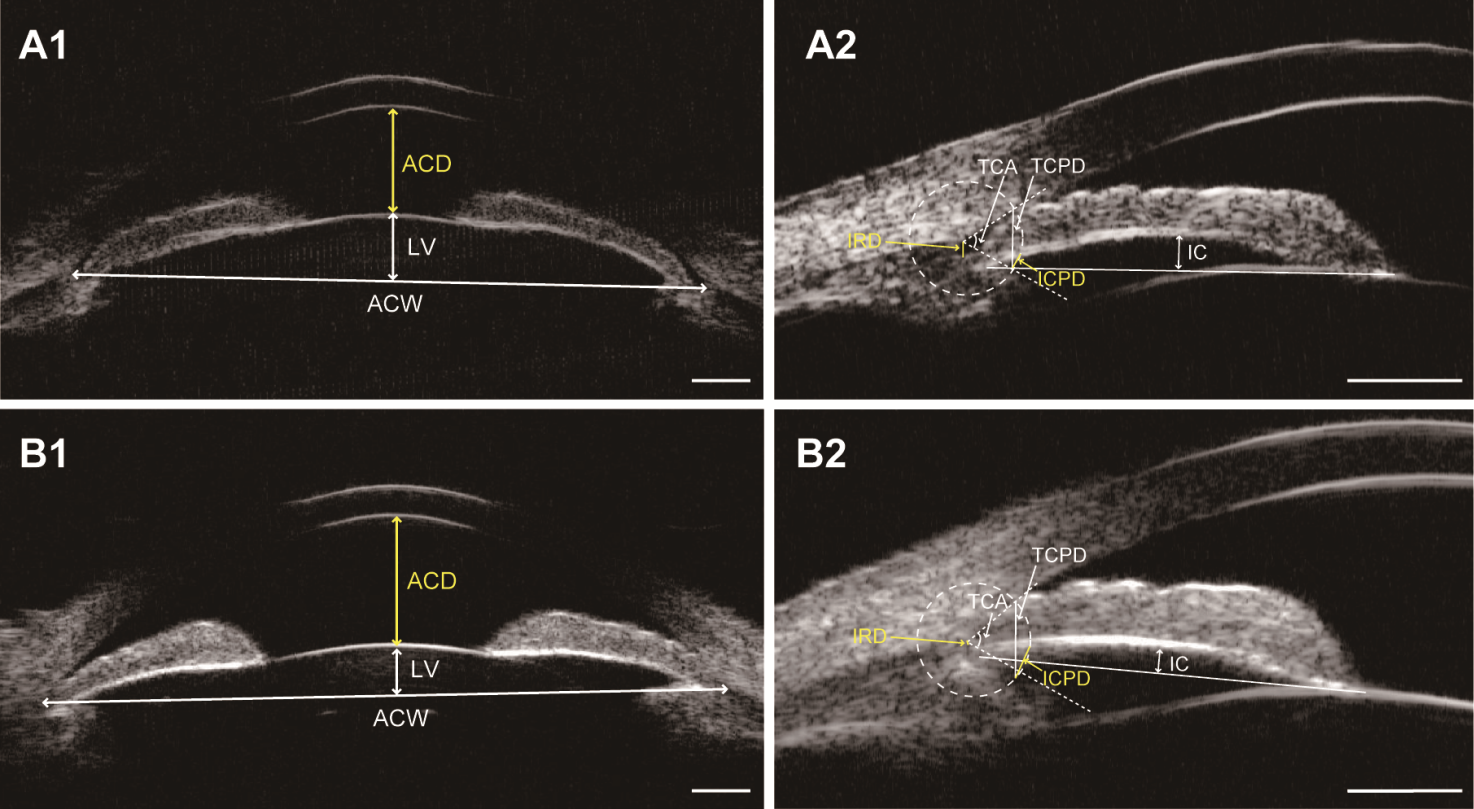
Ultrasound biomicroscopy images of two patients (patient A and B). A, The fellow eye of a patient with acute primary angle closure (APAC). B, The fellow eye of a patient with chronic primary angle closure (CPAC). Note that the fellow eye of APAC has smaller anterior segment dimensions (anterior chamber depth [ACD] and anterior chamber width [ACW]), higher lens vault (LV) (A1 vs. B1), greater iris curvature (IC), more posterior iris insertion (longer iris root distance [IRD]), and more anteriorly positioned ciliary body (shorter trabecular-ciliary process distance [TCPD] and iris-ciliary process distance [ICPD], and smaller trabecular-ciliary angle [TCA]) (A2 vs. B2). Scale bar: 1mm.

A growing body of research concentrates on comparison of biometric differences between APAC(G) eyes and CPAC(G) eyes or between fellow eyes and affected eyes in APAC(G) to establish the predictors associated with the occurrence of acute attacks [7,8,28–30]. However, anatomic structures in anterior segment would have tremendous morphological change after acute attacks, especially for iris parameters. Therefore, investigating unaffected or milder affected eyes that predispose to APAC(G) or CPAC(G) makes more sense than directly comparing APAC(G) and CPAC(G) eyes, which could show the original anatomic configuration before the diseases develop to the severe stages. To the best of our knowledge, this is the first cross-sectional study to comprehensively compare the differences between fellow eyes of APAC(G) and fellow eyes of CPAC(G) before LPI, rather than post LPI [16]. In our patients, fellow eyes of APAC(G) had smaller anterior segment dimensions (ACD and ACW), higher LV, more posterior iris insertion (IRD), greater IC, and more anteriorly rotated ciliary body (TCPD, ICPD, and TCA) compared with fellow eyes of CPAC(G) (Fig 3). We also found that ACD, ICPD, and IRD were the three most important factors that could differentiate these two kinds of predisposed eyes.

Smaller anterior segment dimensions (such as ACD and ACW) are regarded as risk indicators for angle closure [4]. Numerous studies have proposed that the shallower the ACD, the higher risk for the occurrence of APAC [28,30,31]. Also, the recent study showed that the ACD of fellow eyes with APAC were significantly shallower than that of fellow eyes with CPACG [16], which is consistent with our findings. However, ACW was reported to have no significant differences between APAC and CPAC(G) [7], while our results showed that fellow eyes of APAC(G) had smaller ACW than fellow eyes of CPAC(G). This might be because extensive PAS in CPAC(G) could cause slightly inaccuracy on determination of scleral spurs. Therefore, our findings indicate that the smaller anterior segment dimensions that predispose to APAC include not only ACD, but also ACW.

The extent of the lens located anterior to the chamber angles can be quantified by LV, which has better performance than other parameters of lens, such as lens position (LP, defined as ACD+1/2 LT) and relative lens position (defined as LP/AXL) [2]. In our study, UBM imaging demonstrated that fellow eyes of APAC(G) had greater LV than fellow eyes of CPAC(G) although these two kinds of eyes had no difference in LT. These results are similar to the recent AS-OCT findings of greater LV in fellow eyes of APAC compared with CPACG eyes [7,8,10]. We speculate that the increased LV may push iris anteriorly and aggravate pupillary block, thus leading a “crowded” angle into an acute attack.

Iris-related parameters are also essential to the development of angle closure [25]. We used IC, IRD, and IT500 as three separate variables to quantitatively describe iris characteristics. The degree of IC represents the pressure gradient between posterior and anterior chambers, which can presumably result from pupillary block. Some investigators even regarded IC in UBM images as the criteria for identifying the existence of pupillary block in angle closures [32]. In the present study, we found that IC was greater in fellow eyes of APAC(G) than fellow eyes of CPAC(G), which represents that the degree of pupillary block component is greater in fellow eyes of APAC(G) than fellow eyes of CPAC(G). Since the fellow eyes could represent the affected eyes in the same patient to a certain extent, it suggests that the pupillary block component might play a greater role in the acute attack. This contrasts with the previous study in which APAC eyes and CPACG eyes had similar IC [7]. It is unsurprising because the affected eye of APAC has relatively flatter iris configuration than its fellow eye since the iris shape could dramatically change during the acute attack [29,30]. The insertion location of iris into the ciliary body can be quantified by IRD and shorter IRD suggests a more anterior iris insertion [26]. In a qualitative UBM study reported by Chen et al. [16], fellow eyes of CPACG had more proportion of anterior iris insertion than fellow eyes of APAC, although no statistical significant difference was found between two groups. Consistently, our study demonstrated that fellow eyes of CPAC(G) had significantly shorter IRD than fellow eyes of APAC(G) by using quantitative method, which basically supports the idea that CPAC(G) may tend to have more anterior iris insertion than APAC(G). This is mainly because the mechanisms of these two forms of angle closure might be different. “Creeping closure” has been considered as one of the mechanisms in chronic angle closures [2], so shorter IRD increases the opportunity to gradually contact anterior chamber angles. While iris bombe resulting from pupillary block has been proposed as one of the main mechanisms of acute angle closures [2], so short IRD might not be necessary in the acute attack. With regard to IT500, we did not find significant difference between these two kinds of eyes with light-conditioned UBM, which is different from the previous research showing thinner IT500 in fellow eyes of APAC than that in fellow eyes of CPACG under a dark condition [16]. The discrepant findings may be because the UBM examination was performed after LPI and under a different illumination in that study.

Anteriorly positioned ciliary body is associated with the non-pupillary block mechanism of angle closure [33]. Our study found that fellow eyes of APAC(G) had shorter TCPD and smaller TCA than fellow eyes of CPAC(G), demonstrating that the ciliary body was more anteriorly rotated in fellow eyes of APAC(G), which is in agreement with previous reports that affected eyes of APAC had more anteriorly positioned ciliary body than CPACG eyes [11,12]. On the contrary, Chen et al. [16] noted that fellow eyes of CPACG had more anteriorly rotated ciliary body (smaller scleral-ciliary process angle [SCPA]) than fellow eyes of APAC. This may be largely because the measurement of SCPA depends on operator’s subjective judgement which increases the margin of error for this parameter. Thus, we used TCPD and TCA instead of SCPA in our study. Our results suggest that anteriorly positioned ciliary body could be a cause for angle closure and might play more important role in APAC(G) than in CPAC(G). Furthermore, since choroidal expansion could be another important factor in the progression of APAC [34], anteriorly positioned ciliary body might be the secondary change due to the “push effect” caused by increased intravitreal pressure with choroidal expansion.

Taking all the anatomic factors as well as age and sex into account, in logistic regression analysis, we found that shallower ACD, shorter ICPD, and longer IRD were independently and significantly related with the presence of acute attacks. Among them, ICPD is an indicator associated with both the position of the ciliary body and iris insertion. That is, the more anteriorly rotated the ciliary body is, the smaller the ICPD will be; the more posterior the iris insertion is, the smaller the ICPD will be. Our findings reveal more posterior iris insertion and more anteriorly positioned ciliary body in fellow eyes of APAC(G) than those in fellow eyes of CPAC(G); it is likely the composite effect that makes ICPD more sensitive to differentiate these two kinds of eyes.

The biometric differences between these two kinds of predisposed eyes found in our study may reveal two different forms of angle closure. Greater IC and more posterior iris insertion in fellow eyes of APAC(G) imply that the closures of APAC(G) might start in the vicinity of Schwalbe’s line since the summit of bulging iris is likely to touch the vicinity of Schwalbe’s line earlier than the bottom of the chamber angle, while the closures of CPAC(G) might start from the bottom of the chamber angle due to more anterior iris insertion. Our results actually corresponded to type S and type B angle closure respectively proposed by Sakuma T et al. [35].

One of the limitations of our study was the relatively small sample size. Despite this, there was still sufficient statistical power to detect significant differences between these two types of anatomically predisposed eyes. Secondly, the cross-sectional design made us unable to establish the temporal relationship between changes of anterior segment variables and the presence of APAC(G) or CPAC(G). However, following predisposed eyes without interventions would not be ethically feasible. Therefore, comparing the fellow eyes of unilateral APAC(G) and milder eyes of asymmetric CPAC(G) might be an alternative way to observe the original anatomic differences before the diseases develop. Besides, in order to avoid potential bias from the measurement, patients with extensive PAS were excluded, which made the sample in our research is potentially skewed towards milder forms of the diseases. Lastly, although miotics were not used or discontinued for at least one week before UBM examination, two fellow eyes of APAC(G) and four fellow eyes of CPAC(G) used other antiglaucoma drugs, including β-adrenergic antagonists and carbonic anhydrase inhibitors, which have not been reported to affect the anterior segment morphology. Yet their hidden interference was unknown.

In conclusion, we have detected significant differences in biometric and UBM parameters between fellow eyes of APAC(G) and fellow eyes of CPAC(G). Compared with fellow eyes of CPAC(G), fellow eyes of APAC(G) had smaller anterior segment dimensions, higher LV, more posterior iris insertion, greater IC, and more anteriorly rotated ciliary body. ACD, ICPD, and IRD were the three most important parameters that distinguish these two kinds of predisposed eyes.

## Supporting information

S1 File Raw data of our research. (XLSX)

S2 File STROBE Statement. (DOCX)
